# Dr. Allen E. Bradley

**Published:** 1913-10

**Authors:** 


Dr. Allen E. Bradley (not listed in State Directory) died in Norwich, N. Y., August 26, aged 52.

